# The impact of high temperatures on *Vitis vinifera* cv. Semillon grapevine performance and berry ripening

**DOI:** 10.3389/fpls.2013.00491

**Published:** 2013-12-03

**Authors:** Dennis H. Greer, Mark M. Weedon

**Affiliations:** ^1^School of Agricultural and Wine Sciences, Charles Sturt UniversityWagga Wagga, NSW, Australia; ^2^National Wine and Grape Industry Centre, Charles Sturt UniversityWagga Wagga, NSW, Australia

**Keywords:** photosynthesis, rate of ripening, soluble solids concentration, stomatal conductance, transpiration, yield

## Abstract

The heat event that occurred in many parts of Australia in 2009 was the worst on record for the past decade, with air temperatures exceeding 40^°^C for 14 days. Our aim was to assess the impacts of this heat event on vine performance, including ripening, yield, and gas exchange of *Vitis vinifera* cv. Semillon grown in a Riverina vineyard. To assess the affect of high temperatures on Semillon grapevines, the vines were covered with a protective layer to reduce radiant heating and were compared with vines exposed to ambient conditions. The heat event had major effects on ripening; reducing the rate of ripening by 50% and delaying harvest ripeness and causing a high incidence of berry shrivel and sunburn. Yield was not affected. Photosynthesis was reduced 35% by the heat event while transpiration increased nearly threefold and was accounted for by increased stomatal conductance. The conclusion of this study was that heat events delayed ripening in Semillon berries and caused a significant reduction in berry quality. Strategies to minimize the radiant load during heat events are required and this study has confirmed a protective layer can reduce canopy temperatures and enhance berry quality.

## INTRODUCTION

High temperatures are a common occurrence in grape growing regions of Australia. Temperatures exceeding 40°C can be sustained over several days. Recent high temperatures have occurred in Australia in the summers of 2006 and 2009 and in both instances the high temperatures persisted for more than 5 days (Australian Bureau of Meteorology, 2011). Such high temperatures can occur as early as the time of flowering right through to harvest (Gladstones, 1992). Crop losses can occur when high temperatures affect flowering but also later in the season can slow growth of berries and impede sugar accumulation (Greer and Weston, 2010) and thus delay harvest, reduce yields, and compromise berry composition. By contrast, Soar et al. (2009), using chambers to increase air temperatures to about 40°C around Shiraz canopies showed no impact of the increased temperatures on berry sugar ripening. Thus, there appears to be varietal differences in the response of vines to high temperatures and radiation intensity.

The effect of high temperatures on a number of other grapevine varieties has, nevertheless, been well documented. For example, Kliewer and colleagues (Kliewer and Lider, 1968; Kliewer, 1977; Matsui et al., 1986; Sepúlveda and Kliewer, 1986; Sepúlveda et al., 1986) have extensively investigated high temperature effects on such cultivars as Emperor, Thompson Seedless, Napa Gamay, Cabernet Sauvignon, Chenin Blanc, and Chardonnay. Of the many effects of high temperatures, perhaps the most important for growers and winemakers are reduced berry development and delayed ripening. More recently, Greer and Weston (2010) showed similar effects in potted Semillon grapes when vines were exposed to high temperatures. Their study also revealed that susceptibility to high temperatures was dependent on the stage of bunch/berry development, with flowering, veraison, and mid-ripening being highly susceptible stages while the fruit set stage was highly tolerant of high temperatures. As also documented by Greer and Weston (2010), most studies of convective heat (mass air heating) impacts on grapevines have been conducted in controlled environments while most vineyard studies have mostly examined radiative (direct sun exposure) effects (Crippen and Morrison, 1986; Price et al., 1995; Bergqvist et al., 2001). To date, there appears to be a paucity of knowledge of the performance of common grape varieties in vineyard conditions to the high temperatures that occur during the summer advective air heating that occurs in hot climates.

Shade cloth covering whole vines has been used as a means of ameliorating canopy temperatures of grapevines. For example, Sangiovese vines covered with 40 and 70% shade cloth (Cartechini and Palliotti, 1995) caused average within-canopy temperatures to decline by just over 2°C. Similarly, Morrison and Noble (1990) used shade covering over whole vines to examine fruit and wine sensory properties although the details of the shade and the impact on temperatures were not given. By contrast, thin net and plastic films covering Italia grapevines reduced midday temperatures by about 6°C below air temperature (Rana et al., 2004). Thus, there are indications that covering vines with shade cloth can be an effective means of reducing canopy temperatures. Millar (1972) has also shown shading by foliage alone can reduce Muscat of Alexandria berry temperatures by above 10°C and similar results have been shown with Merlot berries by Spayd et al. (2002).

However, shading of grape bunches is also known to have an effect on berry composition and almost always reduces sugar concentrations and delays ripening (Jackson and Lombard, 1993). On the other hand, complete darkening of Thompson Seedless grapes slightly increased soluble solids (SS) concentration as well as total acidity (Kliewer and Antcliff, 1970). By contrast, shading of Shiraz bunches had no effect on sugar accumulation but did reduce flavonol concentrations in the skin (Downey et al., 2004). A similar observation was made with shaded Merlot grapes (Spayd et al., 2002). Shading of Cabernet Sauvignon bunches also had no affect on sugar or acid accumulation but anthocyanins and soluble phenolics were reduced in comparison with exposed bunches (Morrison and Noble, 1990). On the other hand, when Sangiovese grapevines were subjected to increasing shade, SS concentration increased and titratable acidity decreased (Cartechini and Palliotti, 1995). Thus, the berry compositional response to shading appeared to depend on the particular variety.

This project was part of a larger study into the effects of light and temperature on Semillon grapevine performance (Greer et al., 2010, 2011; Greer, 2012). The objective of this study was to investigate the effect of high temperatures on vine performance and berry ripening in Semillon grapevines growing in vineyard conditions.

## MATERIALS AND METHODS

### FIELD SITE

This study was undertaken on a commercial vineyard in the Murrumbidgee Irrigation Area in NSW, Australia (lat. 34.25°S, long. 146.19°E, 129 m asl) over the 2008/09 growing season. The 6 year old *Vitis vinifera* cv. Semillon (accession DA16162) vines were grown on own roots. Rows were orientated North–South at 1.8 m spacing between vines and 3.5 m between the rows and the vines grown on a vertical shoot positioned trellis, with shoots lifted in late spring. The vines were drip irrigated at 2.4 L h^-^^1^ for 12 h per week until ripening commenced and then increased to 24 h per week through to harvest. Nutrition was supplied through the dripper system. Midday water potentials measured in midsummer averaged -1.6 ± 0.1 MPa on both treatments and otherwise there were no signs of water stress. The site is characterized by long-term average monthly mean maximum and minimum temperatures of 30.8/15.0, 32.6/16.6, and 32.1/17.2°C from December to February (Greer and Weedon, 2012a). The vapor pressure deficit (VPD) over the summer averaged 3.1 ± 0.2 kPa.

### TREATMENT SYSTEM

Two panels of vines were selected as fully exposed vines and two further panels covered with a protective layer of 70% neutral density shade cloth (Shade Australia, Sydney, NSW, Australia) were selected to serve as protected vines with reduced canopy temperatures to compare the effects of high temperatures. The experimental treatments were replicated once along the same row.

### TEMPERATURE MEASUREMENTS

On each side (East and West) of the center vine in a panel of each treatment was placed an infrared temperature sensor (IRRP, Apogee, Logan, UT, USA) at 1.2 m height above the ground and 0.3 m from the canopy and pointed directly at the mid canopy. The sensors were connected to a data logger (CR1000, Campbell Scientific Australia, Townsville, QLD, Australia) and hourly average temperatures recorded each day. Air temperatures and humidity (HMP50, Vaisala, Helsinki, Finland) in a white 8 × 15 cm cylindrical screen with five separated plates each 1.2 cm apart and placed 500 mm above the canopy were also measured. Temperature of four bunches per treatment and replicate, two each on representative bunches on the eastern and western sides of the canopy were measured with thermocouples. These were inserted into the bunch shortly after flowering and berries were allowed to grow around the thermocouple. Photon flux densities (PFDs) in each treatment were also determined at hourly intervals with quantum sensors (LI190s, LiCor, Lincoln, NB, USA) located 500 mm above the canopies.

### GAS EXCHANGE

Gas exchange was measured using an open gas exchange system (LCA4, Analytical Development Company, Hoddesdon, UK). All the leaves on two shoots on each of six vines in each treatment were measured at about weekly intervals. An increasing number of leaves were measured as the season progressed. All measurements occurred between 9 am and 4 pm. PFD and leaf temperatures were measured simultaneously with a quantum sensor and thermistor attached to the leaf cuvette of the gas exchange system.

### BERRY RIPENING

Three berries were sampled from the top, middle, and bottom segments of a selected bunch of each of six shoot on each vine at regular intervals through the late growing season. The berries were removed and total percentage SS of each berry measured with a digital refractometer (PR-101, Atago, Tokyo, Japan) in the vineyard.

### YIELD AND BERRY ATTRIBUTES AT HARVEST

At harvest, all bunches on the vines in each treatment were counted and the total bunch fresh weight per vine recorded. These bunches were then taken to the laboratory and assessed for numbers of damaged (sunburned or shriveled) berries, bunch fresh weight, berry diameter on three berries of each bunch and then each bunch was dried at 60°C for 2 weeks to determine dry weight. Wine was then made for each treatment and replicate with all bunches destemmed, crushed, and fermented at 16°C for 5 days then stored at 4°C for 30 days. The wine was then racked, filtered, bottled, and assessed for acidity, alcohol, and phenolic content using the procedures of Iland et al. (2004).

### DATA ANALYSIS

All data were analyzed using generalized linear models with SAS Ver. 9.13 (SAS Institute, Cary, NC, USA) and least squares means and standard errors determined. All data were analyzed using a randomized design and statistical significance assessed at the 5% level.

## RESULTS

### FREQUENCY OF HIGH TEMPERATURES IN THE REGION

**FIGURE 1:**
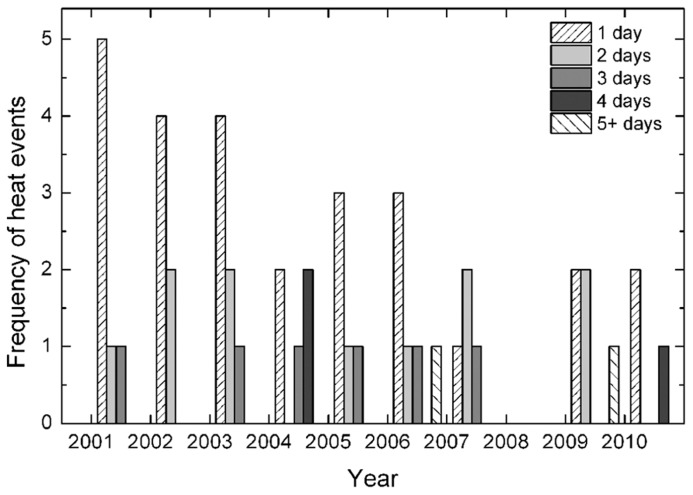
**Frequency of concurrent high temperatures (daily maximum temperatures exceeding 40°C) for 1–5 or more days (as indicated) during the summer months from December to February at the Griffith Airport for the period from 2001 to 2010**. These data were provided by the Australian Bureau of Meteorology, 2011 for Station Number 075041.

Between 2001 and 2010 at the nearby Griffith, NSW Airport, daily maximum air temperatures above 40°C during the main part of the growing season (December to February) occurred frequently (**Figure 1**) at an average of 2.5 single-day events per year. Two concurrent days of temperatures above 40°C were also common, occurring twice in several years but averaged only just over one occasion per year throughout the 10 years. High temperatures lasting three days also occurred in 60% of the years but rarely more than once during the growing season. More sustained high temperatures were also relatively rare, though a 4-day event occurred twice in the 2004 growing season and, more rarely still, a 7-day event occurred in 2006. The high temperatures of the 2009 growing season were very unusual within the decade in that temperatures above 40°C persisted for 14 days in a row.

### AIR AND CANOPY TEMPERATURES AT THE VINEYARD

**FIGURE 2:**
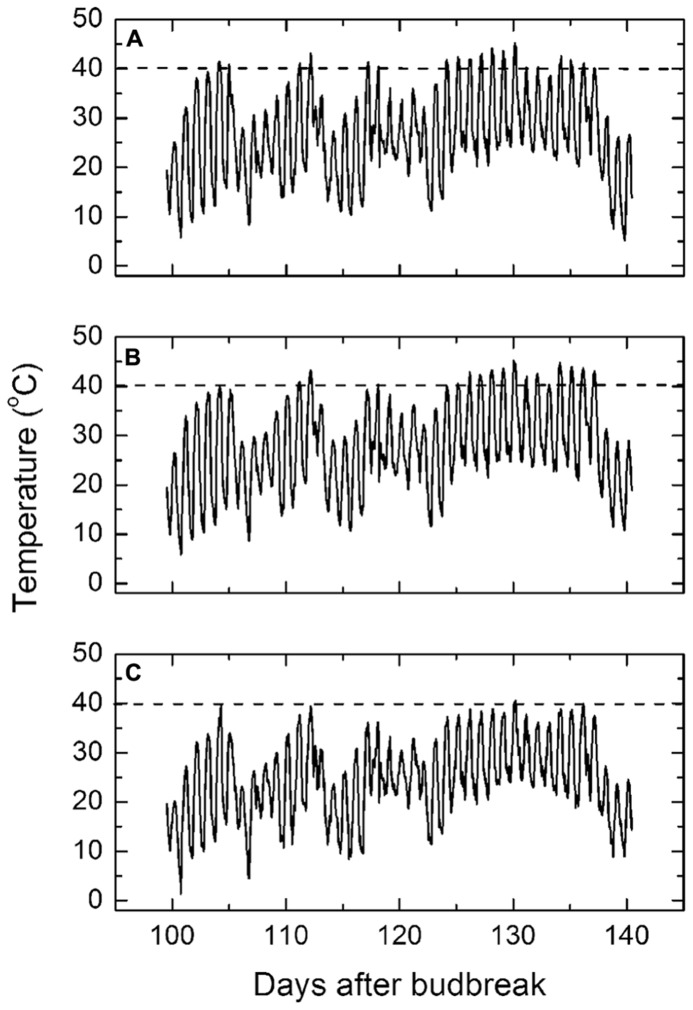
**(A–C)** Mean daily maximum air temperatures **(A)**, exposed canopy temperatures **(B),** and protected canopy temperatures **(C)** from January 1, 2009 to February 10, 2009. The dotted line indicates the 40°C temperature to indicate when the temperatures exceeded this threshold.

The average hourly ambient air temperatures in the vineyard during each day of the mid to late stage of the growing season (**Figure 2A**) reached upward of 40°C on several occasions before the sustained high temperature period occurred, starting on the 26th January and lasting until the 8th February. The average hourly air temperature during this period peaked at 45°C. Thereafter, the maximum daily air temperature dropped abruptly to around 30°C through to harvest.

The average hourly diurnal canopy temperatures of the western side of the exposed vines followed the same pattern of air temperatures throughout the growing season (**Figure 2B**), except that during the sustained high temperature period, the canopy temperatures were up to 2°C warmer than air temperature at the hottest time of the day. By contrast, the average hourly temperatures on the western side of the control vines remained mostly below 40°C (**Figure 2C**), except on the very hottest day when the canopy temperature reached 40.6°C. Thus, the protective layer reduced the canopy temperature by an average of 4.6 ± 0.1°C throughout each day of the high temperatures although the maximum cooling effect often exceeded over 6°C. Otherwise the pattern of canopy temperatures throughout the growing season mirrored air temperature, though offset by 4–5°C. Bunch temperatures followed a similar pattern in all cases and, therefore, not presented.

Maximum PFDs in the exposed canopies were above 1000 μmol m^-^^2^ s^-^^1^ for most of the growing season and above 1500 μmol m^-^^2^ s^-^^1^ in the later part of the growing season. For the protected vines, the maximum PFD was 400 μmol m^-^^2^ s^-^^1^. The VPDs in each treatment averaged 1.9 ± 0.5 kPa in both treatments.

### GAS EXCHANGE

#### Photosynthesis

**FIGURE 3:**
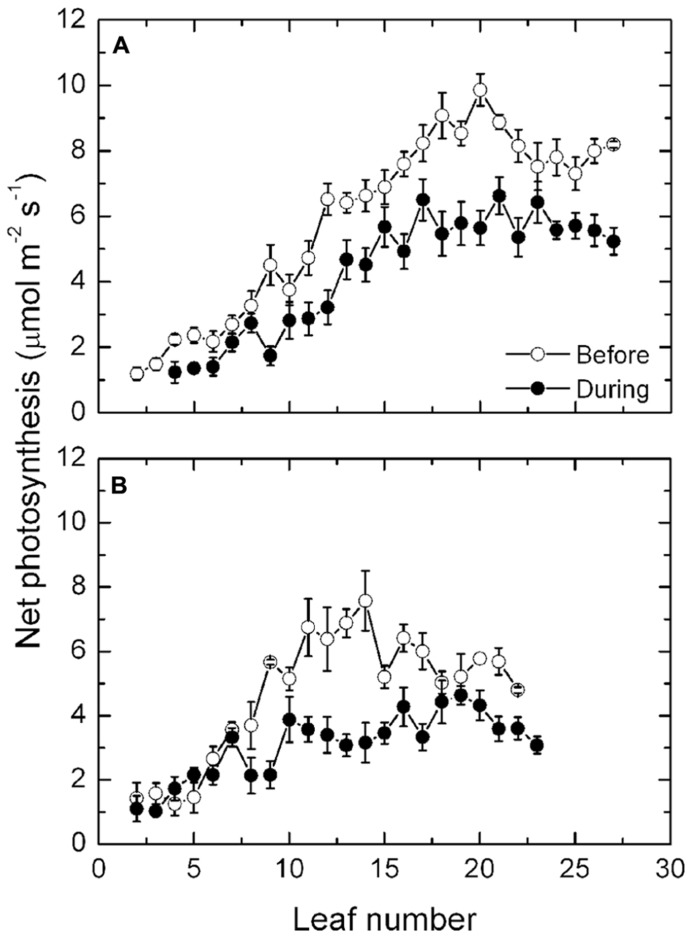
**(A,B)** Net photosynthesis (mean ± SE, *N* = 12) at different leaf positions along the shoot of Semillon vines either prior to (7th January, 104 DAB) or on one day (4th February, 132 DAB) during the 14-day period of high temperatures as indicated. The vines were grown in an irrigated vineyard **(A)** without protection and **(B)** with protection provided by shade cover over the vines.

Prior to the high temperatures occurring, mean leaf photosynthesis along the shoot of the exposed vines increased from about 2 μmol m^-^^2^ s^-^^1^ in the basal leaves to a maximum rate of 10 μmol m^-^^2^ s^-^^1^ at about leaf 20. Thereafter, rates declined slightly in the remaining younger leaves of the shoot toward the shoot apex (**Figure 3A**). During the period of high temperatures, rates of photosynthesis in leaves from about leaf position 7 onward all declined significantly, though the effect was greatest at leaf positions 16–24, where rates varied between 5 and 6 μmol m^-^^2^ s^-^^1^. Thus, a 30–50% reduction in photosynthesis occurred as a consequence of the high temperatures and radiation. A similar pattern along the shoot occurred in the protected vines (**Figure 3B**), with rates of photosynthesis declining markedly during the heat event. Overall, the rates of photosynthesis were lower in the protected compared with the fully exposed vines.

#### Transpiration

**FIGURE 4:**
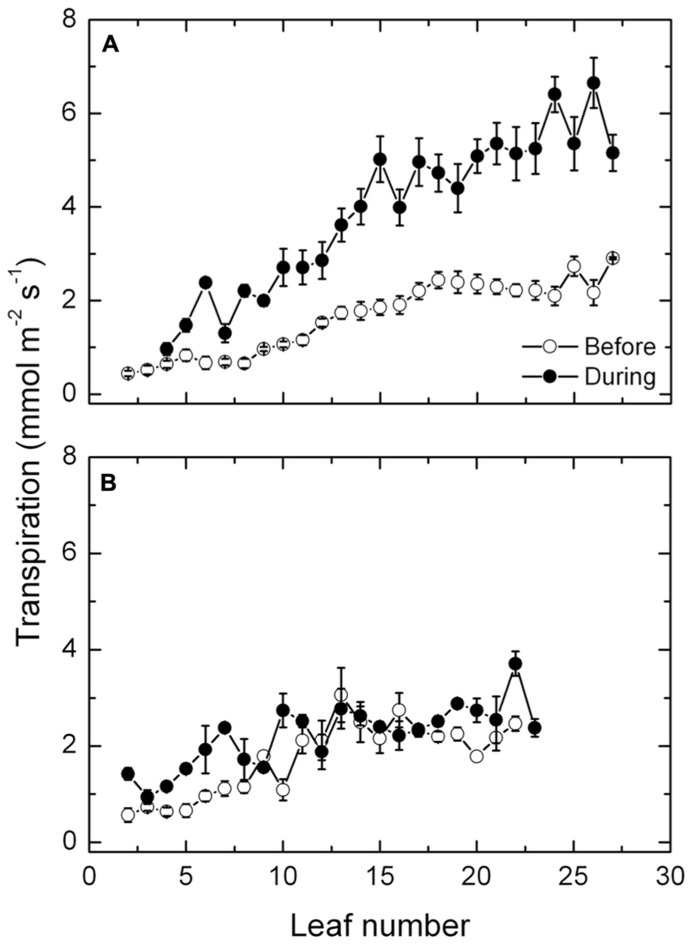
**(A,B)** Stomatal conductance (mean ± SE, *N* = 12) at different leaf positions along the shoot of Semillon vines either prior to (7th January, 104 DAB) or one day (4th February, 132 DAB) during the 14-day period of high temperatures as indicated. The vines were grown in an irrigated vineyard **(A)** without protection and **(B)** with protection provided by shade cover over the vines.

Mean transpiration rates along the shoot of the exposed vines increased steadily from 1 mmol m^-^^2^ s^-^^1^ in the basal leaves to about 3 mmol m^-^^2^ s^-^^1^ in the youngest leaves near the shoot apex, prior to the heat event (**Figure 4A**). During the high temperatures, the same pattern occurred except that the transpiration rates increased progressively along the shoot and markedly, up to about 6 mmol m^-^^2^ s^-^^1^. By contrast, the high temperatures had no such effect on transpiration in the protected vines (**Figure 4B**), as rates of transpiration along the shoot did not differ much before and during the high temperatures. Furthermore, rates of transpiration in the protected vines prior to the high temperatures did not differ significantly from the rates of the exposed vines.

#### Stomatal conductance

**FIGURE 5:**
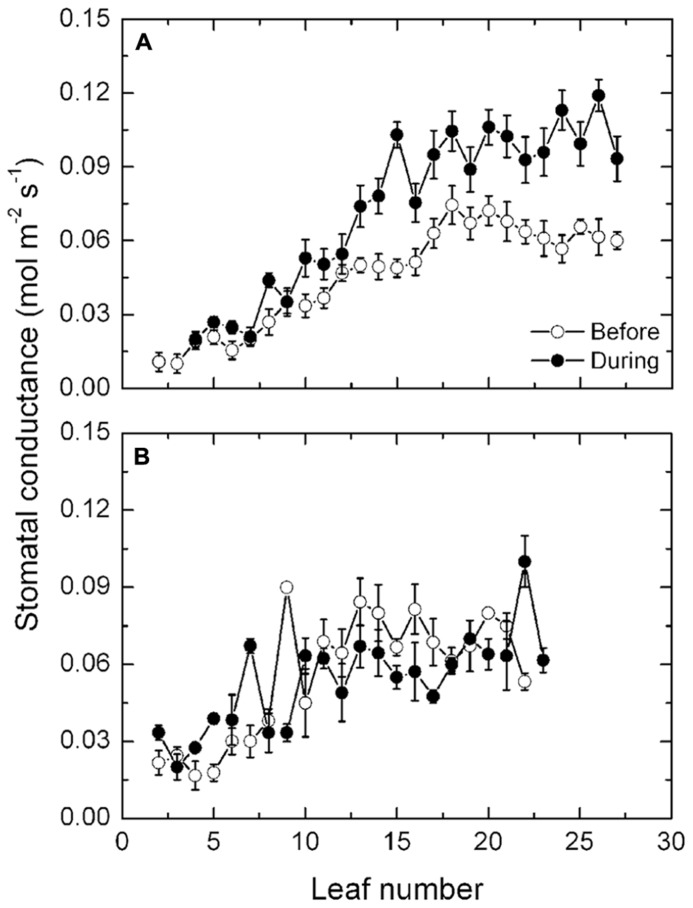
**(A,B)** Transpiration rates (mean ± SE, *N* = 12) at different leaf positions along the shoot of Semillon vines either prior to (7th January, 104 DAB) or on one day (4th February, 132 DAB) during the 14-day period of high temperatures as indicated. The vines were grown in an irrigated vineyard **(A)** without protection and **(B)** with protection provided by shade cover over the vines.

In keeping with transpiration, mean stomatal conductances increased progressively along the shoots of the exposed vines prior to the high temperatures from 0.02 mol m^-^^2^ s^-^^1^ in basal leaves to 0.06 mol m^-^^2^ s^-^^1^ in apical leaves (**Figure 5A**). High temperatures had no consistent effect on the stomatal conductances of the leaves in the lower half of the shoot but from about leaf position 10 onward, stomatal conductance increased markedly during the high temperatures to about 0.1 mol m^-^^2^ s^-^^1^, that is about 66% higher. Again, with the protected vines (**Figure 5B**), there was no effect of the high temperatures on stomatal conductance in any of the leaves along the shoot and the conductances were comparable with those in the exposed vines prior to the high temperatures.

#### Bunch sugar ripening

**FIGURE 6:**
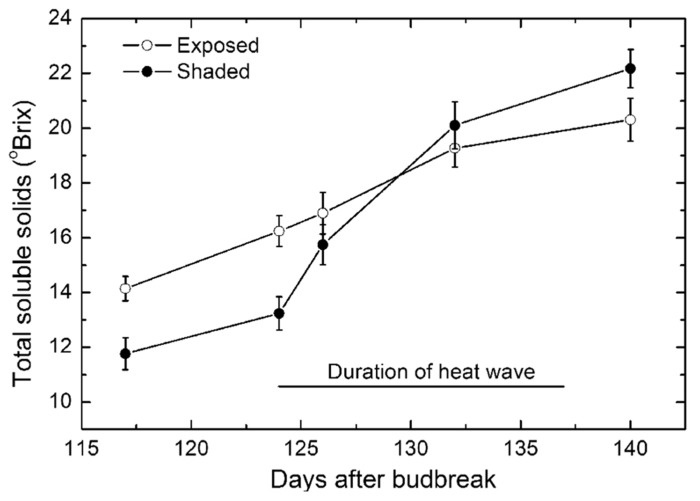
**Changes in soluble solids (mean ± SE, *N* = 36) of Semillon berries during the late stage of ripening for bunches on vines that were not protected and exposed to the full ambient conditions (open symbols) compared with protected vines that were fully protected from the high radiant load (closed symbols) through shade covering. ** The duration of the 14-day period of high temperatures during the ripening period is indicated by the solid line. Note that the sunburned and shriveled berries were excluded from these data.

Just prior to the high temperatures occurring, bunches on the protected vines had total SS of 12 ^o^Brix while bunches on the exposed vines were somewhat riper at 14 ^o^Brix (**Figure 6**). The ripening of the bunches on the both exposed and protected vines ripened at a near linear rate until the start of the high temperature period when ripening in protected bunches increased sharply and continued to become significantly riper than the bunches on the exposed vines. From the early to mid stage of the high temperatures, ripening in the exposed vines appeared to continue more or less unabated until 8–10 days before harvest when ripening slowed down sharply. There was also a decline in rate of ripening for bunches on protected vines but the change was much smaller. Thus at harvest, there were clear and significant differences in ripeness of the Semillon berries in the two treatments.

During the period of high temperatures, the rate of ripening in the exposed vines averaged 0.38 ± 0.01 ^o^Brix day^-^^1^ (*r*^2^ = 0.99, *P* < 0.01) and for the protected vines averaged 0.85 ± 0.11 ^o^Brix day^-^^1^ (*r*^2^ = 0.96, *P* < 0.05), thus significantly higher.

#### Yield and bunch attributes

There was no significant difference between the two treatments in yield and the average for both was 7.2 ± 0.7 kg vine^-^^1^ (**Table 1**). Similarly, bunch numbers per vine were also not different, and both treatments had 46 bunches vine^-^^1^. However, the mean fresh bunch weights were significantly different, with exposed vines 32% higher in weight than the protected vines. Similar significant differences in bunch dry weights also occurred and were 44% higher in the exposed vines compared with the protected vines.

**Table 1:** Yield and bunch characteristics (Mean ± SE, *N* = 12) of Semillon vines grown in exposed conditions and compared with those of protected vines covered by shade cloth over the 2008/09 growing seasons.

Treatment	Yield (kg)	Bunch number	Bunch fresh weight (g)	Bunch dry weight (g)
Exposed	7.3 ± 1.7	40.7 ± 3.9	209 ± 10	153 ± 8
Control	6.8 ± 0.8	45.8 ± 5.9	158 ± 11	106 ± 9
*P*	ns	ns	0.005	<0.001

*All measurements were conducted at the harvest of bunches on 18th February 2009 and yield and bunch numbers determined in the field and remaining measurements conducted in the laboratory. Bunch weights are total per shoot whereas yield and bunch numbers are per vine. The probability of significant differences (*P*) is also shown (ns – not significant).
*

#### Berry attributes

There were no significant differences between the two treatments for the berry fresh weights (**Table 2**) but bunches on exposed vines were significantly larger, with 40% more berries. However, these bunches also incurred significant amounts of skin damage, with over threefold more shriveled and sunburned berries compared with the protected bunches. In particular, damage of the exposed bunches amounted to 30% of berries whereas in protected bunches only 11% of berries were damaged. This damage also contributed to a significantly over-ripe character with the SS concentration reaching upward of 30 ^o^Brix.

**Table 2:** Berry attributes at harvest (Mean ± SE, *N* = 36) of Semillon bunches from vines grown in exposed conditions and compared with protected bunches covered by shade cloth during the 2008/09 growing season.

Treatment	Berry fresh weight (g)	Berries bunch^-1^	Berry diameter (mm)	Damaged berries	SS Sultana berries (^o^Brix)
Exposed	1.62 ± 0.04	183 ± 7.6	12.6 ± 0.16	45.9 ± 5.9	29.2 ± 0.9
Protected	1.80 ± 0.08	130 ± 8.7	12.7 ± 0.22	14.4 ± 3.3	23.2 ± 0.8
*P*	ns	0.001	ns	0.001	0.005

*The number of damaged berries included those showing severe skin damage and sunburn but did not include those berries described as “sultana” which were shriveled without obvious skin damage. The mean soluble solids (SS) of these berries are included. However, the fresh weights and berry diameters were measured on undamaged berries. The probability of significant differences (*P*) is also shown (ns – not significant).
*

#### Wine attributes

There were only minor differences in wine composition between the two treatments, with a slightly lower pH and higher total acidity in that from the protected vines (**Table 3**). There was no difference in alcohol content but there was a marked reduction in phenolic content in the wine made from protected compared with exposed vines.

**Table 3:** Attributes of acidity, total acidity, alcohol content, and phenolic content in wine (Mean ± SE, *N* = 2) made from Semillon bunches from vines grown in exposed conditions and compared with protected bunches covered by shade cloth during the 2008/09 growing season.

Treatment	pH	TA (gL^-1^)	Alcohol (%v/v)	Total phenolics (au)
Exposed	3.16 ± 0.01	5.7 ± 0.1	11.6 ± 0.2	0.71 ± 0.19
Protected	3.08 ± 0.02	6.2 ± 0.2	11.5 ± 0.1	0.11 ± 0.08
*P*	0.035	ns	ns	0.015

*The probability of significant differences (*P*) is also shown (ns – not significant).
*

## DISCUSSION

The high temperatures occurring in the 2008/09 growing season persisted for 14 days, with air and canopy temperatures exceeding 40°C. In comparison with previous growing seasons in the region of study, the high temperatures were extreme in duration. Average monthly maximum temperatures around 40°C are common in this region, particularly from December to February (Gladstones, 1992), however, the duration of high temperatures appears to rarely have been reported. For example, Soar et al. (2008) assessed the number of days above 39.9°C in several locations around Australia and showed the frequency to be less than 2 days.

It was notable that the high temperatures occurred during the post-veraison stage of ripening of the Semillon vines when the sugar accumulation process is most active (Radler, 1965; Morrison and Noble, 1990). The ripening of the bunches of protected vines was comparable with the rapid rate occurring at this time in other varieties (Rogiers et al., 2006; Yamane et al., 2006). The impact of the high temperatures on the ripening process of bunches of the exposed vines was, therefore, relatively strong, with the rate of increase in SS concentration reduced by more than 50% compared with the protected vines. High temperatures occurring in the pre-veraison stage in berry development can have a lasting impact on sugar accumulation in the post-veraison stage (Matsui et al., 1986; Sepúlveda and Kliewer, 1986) while in other studies (Soar et al., 2009) high temperatures had no effect on ripening. Similarly, Buttrose et al. (1971) found no marked effect of temperature on ripening of Cabernet Sauvignon berries. However, Greer and Weston (2010) have shown with potted Semillon vines that high temperatures applied at veraison and at mid-ripening caused a significant reduction in sugar accumulation, consistent with the current study. There are several sugar transporters and sucrose metabolic enzymes involved in sucrose loading into the berry (Agasse et al., 2009) and these high temperatures are likely to have had an inhibitory effect on these metabolic processes. There are certainly genes in the berries that influence the metabolism of berry ripening that are inactivated by high temperature exposures (Pillet et al., 2012).

Berry growth is also sensitive to high temperatures as shown by Matsui et al. (1986) with Thompson Seedless and Napa Gamay grape berry diameter expansion slowed down when treated at high temperatures. However, although berry expansion was not followed in the present study, the berry diameters at harvest were similar (12.7 mm) between exposed and protected bunches. By comparison at the 2007/08 growing season harvest, comparable berry diameters were 13.2 ± 0.2 and 12.8 ± 0.1 mm for exposed and protected bunches (Greer, unpublished data), thus, no difference for the protected berries but smaller berries for the exposed bunches in the 2008/09 growing season. Similarly, Greer and Weston (2010) have shown Semillon berry diameters can reach 13.7 mm. All this suggested berry expansion of the exposed Semillon vines was affected by the high temperatures, consistent with the effect on the other cultivars. However, the high temperatures and radiation intensity did have another major effect on the quality of berries, particularly on the western side of the exposed vines. Some 30% of berries in these bunches showed severe visible symptoms of shrinkage to raisin-like berries as well as sunburn (Greer et al., 2006) but no disease symptoms were evident. By contrast, bunches on the protected vines had much fewer berries affected with these symptoms, less than 10% of bunches, and generally the symptoms were much less severe. Thus, the high temperatures and irradiance had severe effects on the ripening process, berry growth, and skin appearance and, hence, overall berry quality.

Greer and Weston (2010) have shown previously that high temperature-induced reductions in sugar accumulation in Semillon berries were attributable to a sustained reduction in photosynthesis over about 12 days after the high temperature treatment. Their data revealed that insufficient carbon was available for the berries to continue ripening during and for several days after exposure to high temperatures. Rates of photosynthesis in the field-grown vines also declined during the period of high temperatures, both in protected and exposed vines, although proportionately more so in the exposed vines. This is highly consistent with the temperature-dependency of photosynthesis of Semillon leaves which decreases markedly above 35°C (Greer and Weedon, 2012b). However, because only SS concentrations were measured and not sugar content, it was not possible to quantify if the reduction in photosynthesis caused a reduction in carbon availability for berry growth in the present study. Nevertheless, the exposed vines still maintained higher rates of photosynthesis compared with the protected vines (Kriedemann, 1968) but this was attributable to the reduced PFD under the shade cover (Greer et al., 2011). This may account for the reduction in bunch biomass accumulation that occurred in the protected compared with the exposed vines. On the other hand, berry fresh weights were higher (*P* = 0.06) in the protected vines while numbers of berries per bunch were significantly higher in the exposed vines and this translated into significantly higher bunch fresh weights in the exposed vines. Despite this, yield per vine was not significantly affected by the high temperatures or the shading.

Semillon vines are characterized by having intrinsically high transpiration rates as well as high stomatal conductance compared with many other common varieties (Rogiers et al., 2009). In the present study, the high temperatures certainly elicited a marked increase in transpiration rates coupled to an increased stomatal conductance, but only in the exposed vines. Transpiration rates exceeded 6 mmol m^-^^2^ s^-^^1^ during the high temperatures, which were markedly higher rates than reported by Rogiers et al. (2009) for the same variety but comparable with the variety Kékfrankos (Zsófi et al., 2009). However, in the Rogiers et al. (2009) study, the Semillon canopy temperatures were about 25°C, well below the canopy temperatures measured during the present study. Thus, it would appear that the exposed Semillon vines were attempting to cool the canopy by latent heat dissipation during the high temperatures. It is noteworthy that the protected vines had no increase in transpiration or in stomatal conductance during the period of high temperatures, even though the vines experienced a relatively high but subcritical temperature regime. The difference in response between the exposed and protected vines was related to the high radiant load on the exposed vines concurrent with the high temperature exposure whereas the protected vines had a dramatically lower radiant load on the canopy.

The stomatal responses to the high temperatures probably accounted for the increased transpiration. However, this is at odds with the earlier study by Greer and Weston (2010) who showed stomatal conductance declined during and after a high temperature event and was accompanied by a decrease in photosynthesis. By contrast, the stomatal response in the present study does not explain the reduction in photosynthesis that occurred during the heat event and cannot, therefore, be ascribed to a stomatal response. The high temperatures probably had an influence on non-stomatal limitations of photosynthesis, particularly carboxylation, and ribulose 1, 5-bisphosphate regeneration. Yamori et al. (2010) with tobacco leaves and Greer and Weedon (2012b) with Semillon leaves, have recently shown leaf photosynthesis to be carboxylation-limited at high temperatures and, therefore, the most likely explanation for the high temperature-induced reduction in photosynthesis. This is especially so given that several reports (Law and Crafts-Brandner, 1999; Haldimann and Feller, 2004; Salvucci and Crafts-Brandner, 2004) suggest the activation state of the enzyme Rubisco is inhibited by high temperatures and consistent with the conclusion above.

## CONCLUSION

The high temperatures in the 2008/09 growing season in the Riverina grape growing region of Australia were sustained for a particularly long duration. The high temperatures and irradiance caused berries to ripening more slowly but also contributed to a severe incidence of sunburn and shrinkage on the berries, especially on the western side of the canopy. A conceptual model accounts for this effect, that of high temperatures inactivating the CO_2_ fixing enzyme Rubisco, thereby reducing photosynthesis and limiting the supply of sugar for transport to the berries. Sugar loading into the berries may also be impacted on by high temperatures, further restricting the supply of sugar to the berry, with the outcome that ripening is slowed down as observed. However, the yield was not significantly affected by the high temperatures although the quality of bunches was reduced through damage incurred by exposure of the berries. Covering vines with shade cloth reduced canopy temperatures significantly and clearly protected the bunches from damage and improved the wine quality and certainly worth more investigation as a practical means of protecting vines from the deleterious effects of high temperature.

## Conflict of Interest Statement

The authors declare that the research was conducted in the absence of any commercial or financial relationships that could be construed as a potential conflict of interest.

## AUTHOR CONTRIBUTIONS

This paper was primarily written by Dennis H. Greer with a contribution by the co-author Mark M. Weedon to the editing and internal review of the manuscript.
